# In Vitro Calcite Crystal Morphology Is Modulated by Otoconial Proteins Otolin-1 and Otoconin-90

**DOI:** 10.1371/journal.pone.0095333

**Published:** 2014-04-18

**Authors:** K. Trent Moreland, Mina Hong, Wenfu Lu, Christopher W. Rowley, David M. Ornitz, James J. De Yoreo, Ruediger Thalmann

**Affiliations:** 1 Department of Otolaryngology, Washington University in St. Louis School of Medicine, St. Louis, Missouri, United States of America; 2 Department of Developmental Biology, Washington University in St. Louis School of Medicine, St. Louis, Missouri, United States of America; 3 The Molecular Foundry, Lawrence Berkeley National Laboratory, Berkeley, California, United States of America; 4 Department of Chemistry, The George Washington University, Washington, D.C., United States of America; Universidad de Granada, Spain

## Abstract

Otoconia are formed embryonically and are instrumental in detecting linear acceleration and gravity. Degeneration and fragmentation of otoconia in elderly patients leads to imbalance resulting in higher frequency of falls that are positively correlated with the incidence of bone fractures and death. In this work we investigate the roles otoconial proteins Otolin-1 and Otoconin 90 (OC90) perform in the formation of otoconia. We demonstrate by rotary shadowing and atomic force microscopy (AFM) experiments that Otolin-1 forms homomeric protein complexes and self-assembled networks supporting the hypothesis that Otolin-1 serves as a scaffold protein of otoconia. Our calcium carbonate crystal growth data demonstrate that Otolin-1 and OC90 modulate in vitro calcite crystal morphology but neither protein is sufficient to produce the shape of otoconia. Coadministration of these proteins produces synergistic effects on crystal morphology that contribute to morphology resembling otoconia.

## Introduction

Biomineralization is an important biological process that provides structural support and a mineral depot to a broad array of living organisms from mollusks to humans. Understanding the critical molecular interactions of the players at the organic and inorganic interface will help answer many fundamental questions regarding processes in the formation of crystallites or mineralized fibrils that are common in biological tissues such as vertebrate bone, tooth dentin [1], and otoconia. Otoconia present in the utricle and saccule of the inner ear are of critical importance for maintenance of equilibrium and orientation in space. While bone and teeth depend upon mineralization by calcium phosphate, otoconia are the only mammalian hard tissue mineralized by calcium carbonate (CaCO_3_). Biomineralization in bone is a dynamic living process continuing throughout the life of the organism. Otoconia however are formed in the embryonic state and mature during a short perinatal period following which they are essentially static until superficial otoconial demineralization begins in midlife [2], [3], [4]. Advancing age accentuates demineralization which progresses to degeneration and fragmentation of otoconia [5]. This leads to loss of equilibrium and a propensity to falling which may result in bone fracture or death. The most common cause of accidental death in elderly patients is falling [6]. Social isolation and cognitive decline are consequences stemming from this pathology. These outcomes constitute a major socioeconomic burden which is becoming more pronounced with the increasing longevity of the human population. Benign positional vertigo (BPV) another pathologic condition also occurs in younger age groups and is due to displacement of otoconia into the semicircular canal system causing inappropriate stimulation of the corresponding receptors for angular acceleration by gravity; consequently minor movements of the head may result in intense vertigo and nausea [7], [8]. BPV is most prevalent between the ages of 60 and 80, particularly in postmenopausal women suffering from osteoporosis [9], [10].

Biomineralization of otoconia requires proximity of ionic and proteinaceous components at the correct time, the correct location, and the correct orientation. The regulation of the underlying biomineralization process of otoconial development is poorly understood. The field of otoconia research has lagged behind other fields of biomineralization research including bone and teeth. Only recently has the most basic information about the role of OC90 in the formation and modulation of calcite crystals been discovered [11].

Several other otoconial proteins have been identified and partially characterized including osteopontin and fetuin-A [12]. Both proteins are well recognized inhibitors of pathologic ectopic apatite formation. The collagenous scaffold protein Otolin-1, previously identified in bony fish [13], was identified and characterized in mammalian otoconia [14], [15]. Otolin-1 is similar to collagen X of mature chondrocytes [16] and is a short chain collagen with an interactive C1q C-terminal domain [14].

In the present study we demonstrate self-assembly of oligomers of recombinant histidine tagged mouse Otolin-1 (rmOtolin-1) by rotary shadowing electron microscopy (rsEM). Moreover our atomic force microscopy studies provide the first evidence for the function of human Otolin-1 revealing that recombinant histidine tagged human Otolin-1 (rhOtolin-1) assembles into a mesh-like network on mica. We demonstrate that recombinant histidine tagged mouse Otoconin-90 (rmOC90) modulates calcite crystal morphology and growth kinetics when added to CaCO_3_ crystal growth solution and this effect is potentiated by co-administration with rhOtolin-1.

## Materials and Methods

### Otolin antibody production

The Rabbit antiserum against mouse Otolin-1 C-terminal was prepared by Sigma Genosys Company. The antigen peptide [H] CFSGFLLYPEETFSKSP[OH] was derived from the C-terminal amino acid sequence of mouse Otolin-1 according to a previous report [15]. For the purification of the antibody, 2 mg antigen peptide was coupled to AminoLink resin following the manufacturer's instructions (Pierce Biotechnology). 20 ml of rabbit anti-mouse Otolin-1 antiserum (Bleed # 3, Rabbit: GN-21, 027) was applied to the packed agarose-coupled antigen peptide column, and eventually eluted with Glycine-HCl buffer at pH 2.5 according to the recommended procedure.

### Construction of rmOtolin-1

Otolin-1 cDNA was amplified by RT-PCR using total RNA purified from E16.5 mouse otocysts. The sequences of upstream and downstream primers were:

Otolin-Forward: 5′ GGC TGC CTG AAT TCG CCA CCA TGT GGA TAT TTT CTT CGC TTT GTG C 3′, Otolin-Flag-Reverse: 5′ GGC TGC CTC TCG AGT TAC TTG TCA TCG TCG TCC TTG TAA TCT GGT GAC TTA CTA AAA GTT TCC TCT GGG 3′, respectively. Otolin-Forward had a Kozak sequence and an EcoRI recognition site. Otolin-Flag-Reverse had an XhoI recognition site and a FLAG tag sequence (underscored) before the stop codon. The fragment was digested with KpnI and XhoI and inserted into pcDNA3.1 (+). The cDNA sequence was confirmed by sequencing.

### Protein expression and purification

For production of recombinant mouse Otolin-1-Flag proteins by human embryonic kidney (HEK293-T) cells, the expression plasmids pcDNA3.1-Otolin-1-Flag were stably transfected into HEK293-T cells with Lipofectamine 2000. The transfected cells were incubated at 37°C and 5% CO_2_ in Dulbecco's Modified Eagle Medium supplemented with 10% Fetal Bovine Serum and 100 U/ml Penicillin and 100 µg/ml Streptomycin. On the following day cells were replaced with fresh medium containing 1 mg/ml G418 and allowed to grow for another two weeks. Neomycin-resistant clones were screened for high level expression of rmOtolin-1 by monoclonal mouse anti-Flag M2 antibodies. HEK293-T cells from two resistant clones with the highest level stable expression of rmOtolin-1 were grown to confluence in DMEM complemented with 5% Fetal Bovine Serum and 500 µg/ml G418, then replaced with CD 293 serum-free medium (Invitrogen) and allowed to grow in T-175 Flasks for another 3 days. The conditioned medium was precipitated by 75% ammonium sulfate, desalted by PD-10 Sephadex G-25 column and adjusted with 10×TBS, pH 7.5 buffer, then applied to Anti-Flag M2 Affinity resin (Sigma) in batch mode. Eventually the protein was eluted by glycine buffer at pH 3.0. Protein concentration was determined by the BCA assay (Pierce). The purity and identity of the proteins were determined by Coomassie staining and Western blot using polyclonal mouse anti-Otolin C-terminal antibody (8 µg/ml) following a 7.5∼10% SDS-polyacrylamide gel electrophoresis.

For production of human Otolin-1-His protein, an OmicsLink Expression-Ready Clone (pReciever-M77 plasmid) containing the gene insert coding for recombinant human Otolin-1 with a C-terminal His tag was purchased from GeneCopoeia and the sequence was confirmed by gene sequencing in the PNACL core at Washington University. Production of rhOtolin-1-His proteins was enabled by the use of the Freestyle Max 293 expression system (Invitrogen) to transfect non-adherent human embryonic kidney (HEK392-F) cells with the expression plasmid containing an insert for human Otolin-1-His per the manufacturer's directions. The transfected cells were incubated at 37°C and 8% CO_2_ for seven days in Freestyle 293 Medium (Invitrogen). The medium containing the secreted protein was extracted from the cell culture and the protein was purified chromatographically by affinity on an AKTA Purifier with a 1 ml HisTrap column (GE Healthcare). Total protein concentration was determined by the Bradford assay (BioRad). The purity and identity of the proteins were determined by Coomassie staining and Western blot using polyclonal mouse anti-Otolin C-terminal antibody (8 µg/ml) following SDS-polyacrylamide gel electrophoresis on an AnyKD (BioRad) gel.

For production of recombinant histidine tagged mouse OC90 protein, the Freestyle Max 293 expression system (Invitrogen) was used to transfect non-adherent human embryonic kidney (HEK392-F) cells with the expression plasmid pcDNA3.1-OC90-His per the manufacturer's directions. The transfected cells were incubated at 37°C and 8% CO_2_ for seven days in Freestyle 293 Medium (Invitrogen). The medium containing the secreted protein was extracted from the cell culture and the protein purified chromatographically by affinity on an AKTA Purifier with a 1 ml HisTrap column (GE Healthcare). Total protein concentration was determined by the Bradford assay (BioRad). Purity and identity of the proteins were determined by Coomassie staining and Western blot using a 1∶1000 dilution of polyclonal mouse anti-His C-terminal antibody (Abcam) following SDS-polyacrylamide gel electrophoresis on an AnyKD (BioRad) gel.

### Crystal growth and scanning electron microscopy

Calcite crystals were grown by slow evaporation of NH_4_HCO_3_ into a CaCl_2_ solution as reported previously [17]. Briefly, calcite crystals were grown on clean 12 mm glass cover slips, placed into wells of a cell culture dish containing the growth solution. The whole set-up was covered with aluminum foil with a few pin holes, and placed into a sealed glass chamber containing NH_4_HCO_3_. Protein aliquots were dissolved in 7.5 mM CaCl_2_ and crystals grown for 48 hours. Crystals were examined with JEOL JSM 6320F Field Emission scanning electron microscopy at 5 KV after sputter coating with gold and palladium to increase the conductivity.

### Atomic Force Microscopy

In-situ measurements on the effect of rmOC90 and rhOtolin-1 on calcite growth were carried out by atomic force microscopy (Digital Instruments J scanner, NanoScope IIIa and V controllers, Veeco Metrology, Inc., Santa Barbara, CA). All measurements were made at room temperature (25°C) and pressure. Freshly cleaved calcite chips of optical-quality Iceland spar (Ward's Scientific, Chihuahua, Mexico) approximately 2 mm×2 mm×1 mm in dimension were mounted in the fluid cell and reactant solutions flowed continuously through the cell. The supersaturated reactant solutions were made by dissolving reagent sodium bicarbonate (NaHCO_3_, Aldrich) and calcium chloride dihydrate (CaCl_2_, Aldrich) into deionized water (18.2 mΩ). The supersaturation, which is defined as 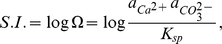
was calculated using the commercial software PHREEQC to be 1.0 [18]. AFM imaging was performed in contact mode using probes consisting of silicon tips on silicon nitride cantilevers from AppNano. The images were used to calculate the speed of atomic step propagation on the calcite (104) surface during exposure to the flowing supersaturated solution for a range of protein concentrations. The pH was controlled at around 7.4 to be close to physiological conditions.

In situ AFM imaging was also used to examine the morphology of rmOC90 and rhOtolin-1 films deposited on mica surfaces. An aliquot of 150 nM solution of each protein in 500 mM KCl was deposited on freshly cleaved mica surface and incubated in the solution for 48 hrs. AFM imaging of the resulting films was performed in tapping mode.

### Raman spectral analysis

Raman spectra were collected on pre-selected crystals of CaCO_3_ with a HoloLab 5000 Raman microprobe spectrometer system (Kaiser Optical Systems, Inc., KOSI), using a 532 nm, frequency-doubled, Nd:YAG solid-state laser as the excitation source, and a holographic grating spectrometer, covering the Raman frequency shift range of ∼−140 to 4300 cm^−1^ at a spectral resolution of 4–5 cm^−1^. The samples were observed and Raman spectra recorded with an Olympus 50X, 0.80 NA air objective which produces a laser beam diameter of ∼3 µm at the focus. The power of the laser beam at the sample was set to ∼13 mW. Spectral data collection times ranged from <1 minute up to 15 minutes depending upon sample size, Raman scattering efficiency, and intensity (if any) of background fluorescence.

### Rotary shadowing electron microscopy

The rmOtolin-1 molecules were visualized by electron microscopy after adsorption on mica, rapid freezing, freeze-drying, and rotary shadowing.

## Results

### rmOtolin-1 and rhOtolin-1 form self assembling networks

**Figure 1:**
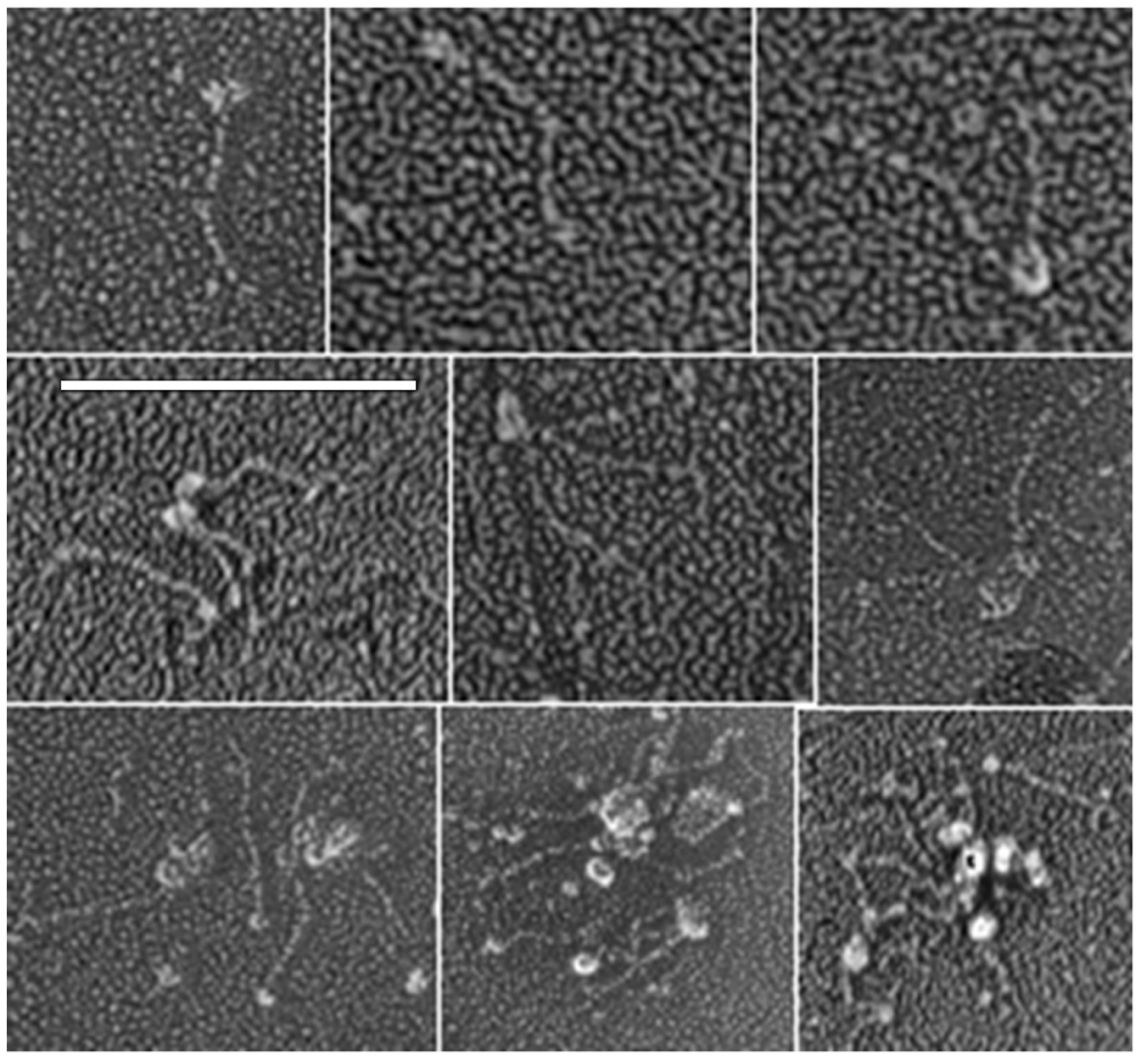
Images of rmOtolin-1 molecules visualized by electron microscopy after rotary shadowing. rmOtolin-1 molecules were adsorbed on mica and visualized by electron microscopy after rapid freezing, freeze-drying, and rotary shadowing. The interaction of the C1q domains is evident and similar to the interactions observed for collagen X by Kwan, et al. [20]. Scale Bar, 100 nm.

**Figure 2:**
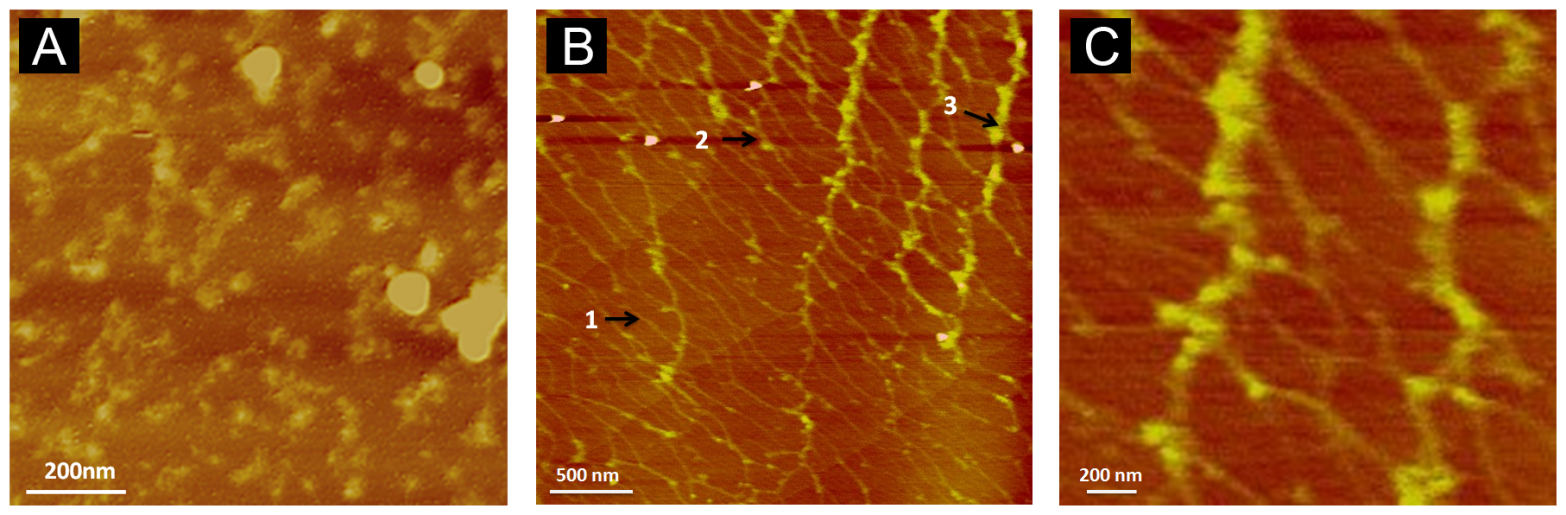
In situ AFM height images (3.0×3.0 µm) exhibiting absorption of rmOC90 and rhOtolin-1 on freshly cleaved mica. (A) rmOC90 sustains cluster formation randomly distributed on a mica surface after 48 hours of incubation. (B) rhOtolin-1 self assembled into a honeycomb network on a mica surface after 48 hours of incubation. rhOtolin-1 fibril height measurements reveal the presence of monomeric (arrow 1), dimeric (arrow 2) and trimeric (arrow 3) components. (C) An enlarged area of the matrix from panel B.

Recombinant, Flag tagged mouse or histidine tagged human Otolin-1 secreted proteins prepared by means of a mammalian expression system (HEK293-T and HEK293-F cell respectively) correspond to an apparent molecular weight of approximately 75 KDa by SDS-PAGE. Western blot analysis utilizing primary antibodies directed against the histidine tag and a C-terminal peptide sequence of mOtolin-1 that shared high amino acid sequence identity with hOtolin-1 established the respective identities of the proteins. Rotary shadowing of rmOtolin-1 reveals a fine tail with a globular head at one end. The tail represents the shorter helical domain of Otolin-1 including the N-terminal domain, whereas the head group corresponds to the highly interactive globular C-terminal C1q domain (Figure 1). The formation of dimers, trimers and multimers of rmOtolin-1 can be attributed to the interaction between C1q domains, as suggested by the aggregation behavior of type X Collagen [19], [20], which tends to form a hexagonal network upon prolonged incubation [20]. Considering that Otolin-1 is a scaffold protein and similar to collagen X, it is reasonable to assume that the fibrillar web observed in the otoconial core would correspond to the hexagonal network. We are currently testing this hypothesis by continuing to study the assembly behavior of rhOtolin-1in vitro. Our preliminary His-tag pull down analysis in addition to the data presented by Deans et al. (2010) has shown clear evidence of an interaction between Flag tagged recombinant mouse Otolin-1 and recombinant mouse OC90-His (data not shown), suggesting that protein-protein interaction could represent the molecular mechanism corresponding to the observed recruitment of rmOC90 by rmOtolin-1. Atomic force microscopy studies of rhOtolin-1 and rmOC90 films on mica demonstrate that rhOtolin-1 self-assembles into filaments that form an interconnected matrix (Figure 2). The honeycomb-like structure of the rhOtolin-1 film may reflect the 3-fold symmetry of the underlying mica lattice. AFM measurements of fibril heights show that fibrils with three distinct thicknesses are formed with average heights of 0.44 nm, 0.93 nm and 1.44 nm, respectively (Figure 2C). The smallest and largest are similar to, but slightly smaller than, the heights of monomeric (0.6 nm) and trimeric (1.5 nm) Type I collagen on mica [21], indicating that the self assembled rhOtolin-1 matrix on mica consists of monomeric, dimeric, and trimeric components.

### rhOtolin-1 and rmOC90 modulate calcite crystal morphology and growth kinetics

**Figure 3:**
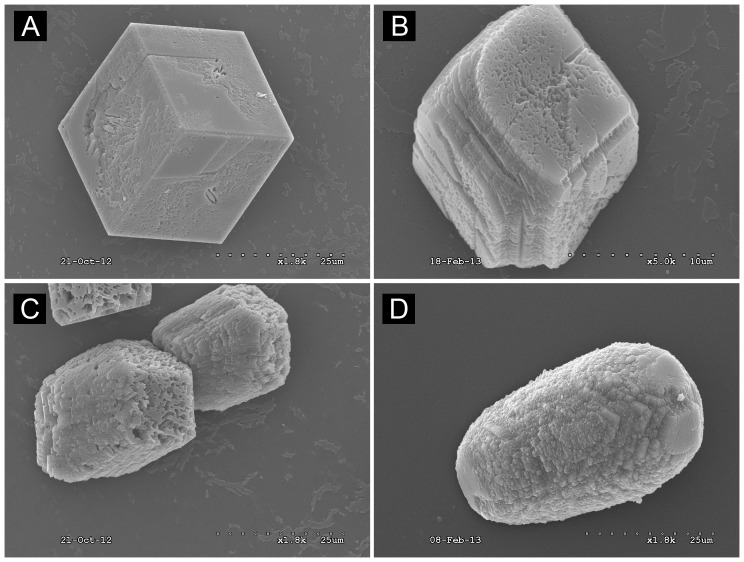
Scanning Electron Microscope Images of calcite modified by rhOtolin-1 alone or in combination with rmOC90. Varying concentrations of rhOtolin-1 and rmOC90 protein, (A) 0 nM rhOtolin-1 and rmOC90; (B) 1000 nM rmOC90; (C) 667 nM rhOtolin-1; (D) 667 nM rhOtolin-1+500 nM rmOC90 were dissolved in 7.5 mM CaCl_2_ growth solution. Crystals were grown for 48 hours by slow evaporation of NH_4_HCO_3_ into growth solution. Crystals were examined with JEOL JSM 6320F Field Emission scanning electron microscopy. Scale Bar, A, C, and D: 25 µm; B: 10 µm.

**Figure 4:**
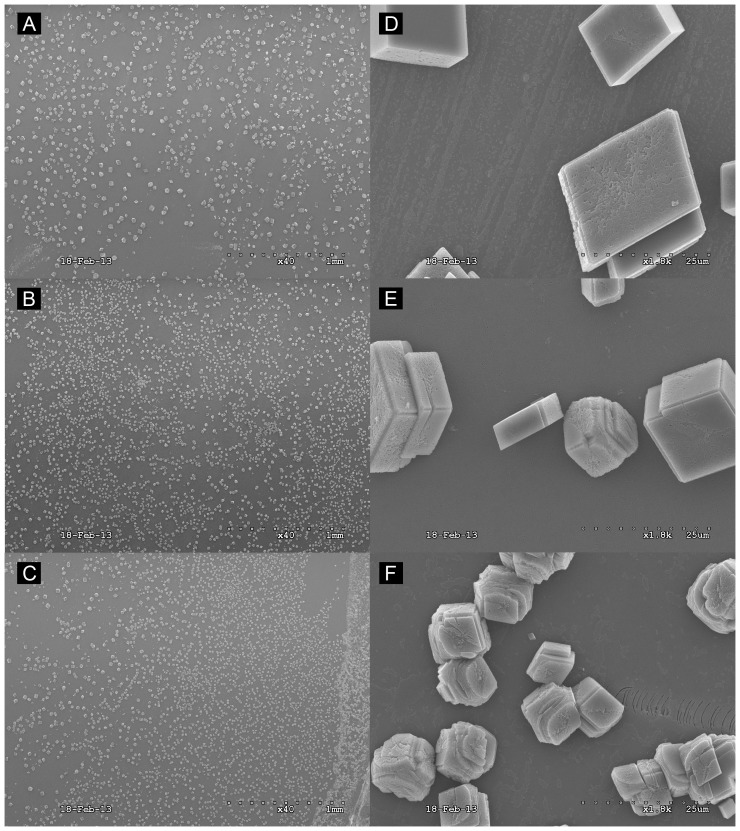
Scanning Electron Microscope Images of protein concentration dependent calcite modification by rmOC90. Varying concentrations of rmOC90 protein, (A and D) 100 nM rmOC90; (B and E) 500 nM rmOC90; (C and F) 1000 nM rmOC90 were dissolved in 7.5 mM CaCl_2_ growth solution. Crystals were grown for 48 hours by slow evaporation of NH_4_HCO_3_ into growth solution. Examination of the crystals was performed with JEOL JSM 6320F Field Emission scanning electron microscopy. Scale Bar, A–C: 1 mm; D–F: 25 µm.

**Figure 5:**
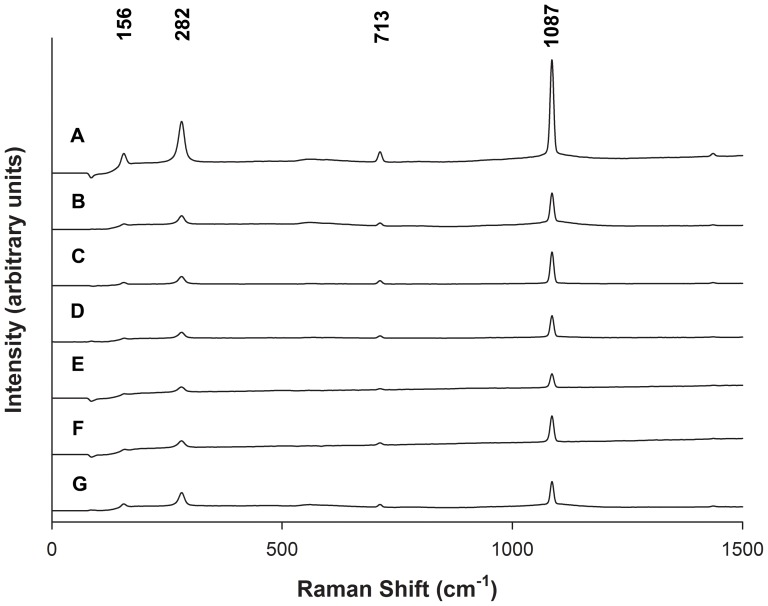
Polymorph determination of calcium carbonate crystals modified by rhOtolin-1 alone or in combination with rmOC90. Varying concentrations of rhOtolin-1 or rmOC90 protein, (A and B) 1000 nM rmOC90; (C and D) 667 nM rhOtolin-1; (E and F) 667 nM rhOtolin-1plus 500 nM rmOC90; (G) pure growth solution were dissolved in 7.5 mM CaCl_2_ growth solution and crystals were grown for 48 hours by slow evaporation of NH_4_HCO_3_ into growth solution. Raman spectra were collected on pre-selected crystals of CaCO_3_ with a HoloLab 5000 Raman microprobe spectrometer system.

The typical morphology of in vitro CaCO_3_ crystals grown on borosilicate showed a cuboidal shape with sharp, well defined edges at the planar interfaces. Upon addition of rhOtolin-1 or rmOC90 to the in vitro CaCO_3_ crystal growth solution we observe significant changes in crystal growth kinetics and crystal morphology (Figure 3A–D). Crystals grown in the presence of rhOtolin-1 alone exhibited rounding of the edges at the planar interface of CaCO_3_ crystals and a tendency toward an elongated or elliptical shape. We also observed an increase in nucleation rate (Figure 4A–B) and rounding of CaCO_3_ crystals in the presence of rmOC90 alone (Figure 3B, Figure 4E–F) in the same protein concentration dependent manner reported by Lu et al. [11]. The morphological changes observed upon the concomitant addition of rhOtolin-1 and rmOC90 to the growth solution are substantially greater than that observed in the presence of either protein alone. The planar interfaces of CaCO_3_ crystals become rounded to a much greater degree and the overall shape of the crystals takes on an elongated and cylindrical morphology (Figure 3D). Raman spectral analysis of CaCO_3_ crystals grown in the presence or absence of rmOC90 or rhOtolin-1 exhibited peaks characteristic of calcite at 156, 282, 713 and 1087 nm (Figure 5) confirming that the crystals consisted of calcite.

**Figure 6:**
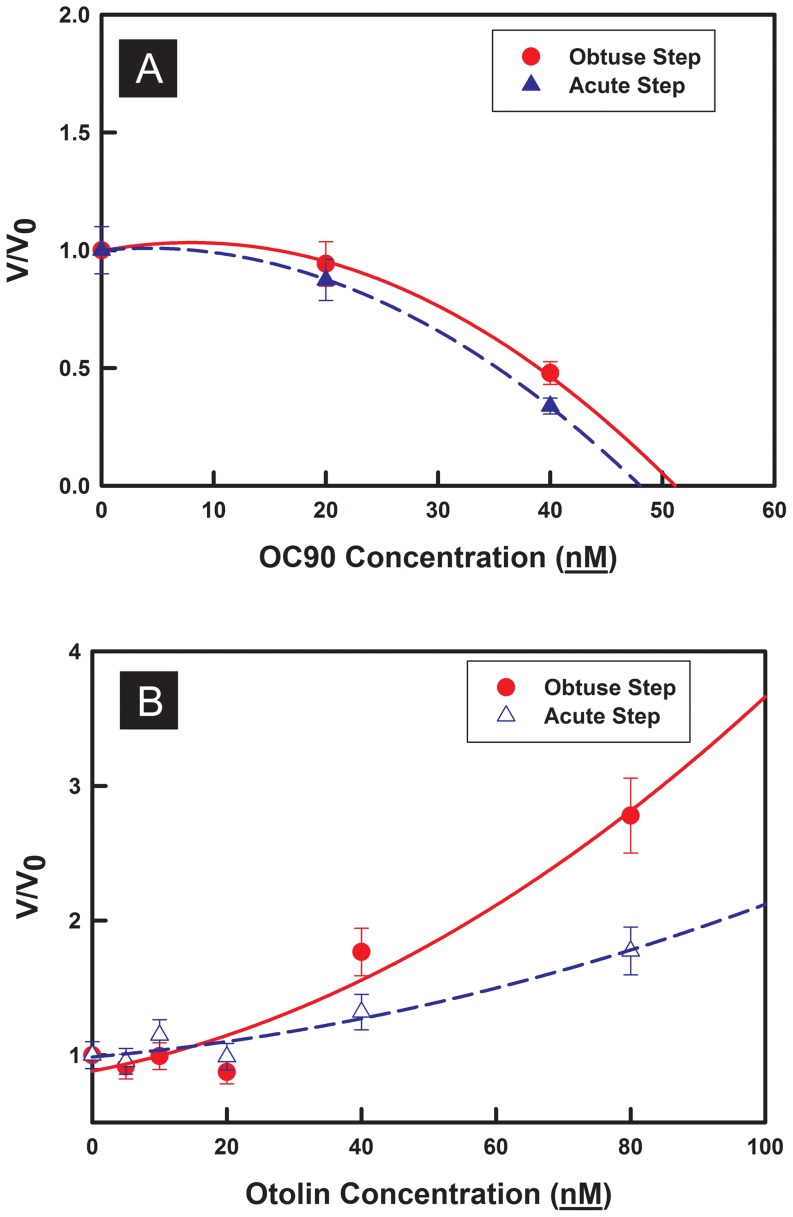
AFM measurements of step propagation rate along both obtuse and acute directions versus rmOC90 or rhOtolin-1 concentration. Normalized step speed rates are shown to exclude the system error between different samples and spirals. (A) Strong inhibition was observed with increasing concentrations of rmOC90. At the concentration of 40 nM the propagation speed was reduced to 30% of the original value. (B) Strong potentiation was observed with increasing concentrations of rhOtolin-1. At 40 nM and 80 nM rhOtolin-1 the propagation speed was increased 177% and 278% of the original value respectively. Error bars (10%) are estimated based on our previous experience with atomic force microscopy measurements [35], [36].

Step speeds on CaCO_3_ crystals grown in the presence of rmOC90 were protein concentration dependent (Figure 6a) for both of the two crystallographically distinct types of steps on the calcite (104) surface, commonly referred to as “obtuse” and “acute” steps. An obvious decrease in the step speed was seen for both steps with increasing rmOC90 concentration. At a protein concentration of only 40 nM step speeds dropped to 30% of the value in the protein-free solution, showing that rmOC90 is a potent inhibitor of calcite growth, with the acute steps more strongly inhibited than the obtuse steps. In contrast, step speeds on CaCO_3_ crystals grown in the presence of rhOtolin-1 exhibited a protein concentration dependent increase (Figure 6b) for both of the two crystallographically distinct types of steps on the calcite (104) surface. The strongest dependence was observed for the obtuse steps. At protein concentrations of only 40 nM and 80 nM rhOtolin-1 step speeds increased to 177% and 278% of the value in the protein-free solution respectively, showing that rhOtolin-1 is a potent promoter of calcite growth.

## Discussion

Immunohistochemical analysis demonstrates that Otolin-1 is localized in both the surrounding otoconial matrix and otoconia. Deans et al. demonstrated the presence of Otolin-1 throughout the otoconial membrane and in the sensory epithelia of the utricle in P2 mice [14]. Yang et al. showed that immunofluorescence labeling of murine Otolin-1 reveals that Otolin-1 is expressed in neonatal B6 mouse sensory epithelium of vestibular utricle and saccule and also in otoconia [16]. This suggests that Otolin-1 functions in a similar manner as other collagen family members in forming fibrous connective tissues and serving as a scaffold for biomineralization. Co-immunoprecipitation studies performed by Deans et al. demonstrate that Otolin-1 does form homomeric and heteromeric protein complexes [14]. Our rotary shadowing data shown here provide the first direct evidence that rmOtolin-1 forms homomeric complexes via interaction of the C1q domains (Figure 1). The rhOtolin-1 morphology observed in our AFM data (Figure 2) supports our conclusion that rhOtolin-1 is a scaffold protein and similar to collagen X [20]. In addition we hypothesize that rhOtolin-1 comprises the cross-linked filaments and isotropic three-dimensional meshwork observed in mature otoconia [22]. Further strengthening this concept is data reported in Andrade et al. which demonstrate that Otolin-1 forms interconnecting fibrils between otoconia that are incorporated into the crystal structure [23].

The interface of inorganic CaCO_3_ with otoconial proteins is poorly understood. Our data presented here demonstrate the morphological modulation of calcite crystals by rmOC90 (Figure 3B, Figure 4). Additionally the data suggest that rhOtolin-1 in solution directly interacts with the inorganic components of otoconia. As evidenced in Figure 3C, the calcite crystal morphology begins to exhibit an elongated shape in the presence of rhOtolin-1 alone. This may represent an important transitional phase of the cuboidal calcite crystal in the early stages of transformation to a cylindrical morphology exhibited by an otoconium. It is important to note that neither rhOtolin-1 nor rmOC90 alone produce the shape of mature otoconia. Our data presented in Figure 3D demonstrate the extreme morphological changes produced in calcite crystals in response to the potentiating effects of co-addition of rhOtolin-1 and rmOC90. It is only in the presence of both proteins that the calcite crystal morphology begins to exhibit a striking resemblance to otoconia [23], [24]. In light of our rotary shadowing data and the data presented by Deans et al. that demonstrate association of Otolin-1 with itself and OC90, it is reasonable to hypothesize that the otoconial precursors observed in Figure 3D result from an rhOtolin-1 scaffold that recruits rmOC90 for nucleation and maturation of calcite crystals into mature otoconia whose shape is due to the synergistic effects on the growth phase by the two proteins.

The combination of the results on crystal morphology, identification of the crystal phase, and the AFM measurements of step speeds on calcite enable us to understand how the combination of Otolin-1 and OC90 can lead to the observed otoconial shape. The three polymorphs of CaCO_3_ (calcite, vaterite, and aragonite) exhibit many morphologies, so it is important to note that we confirmed the identity of the rhOtolin-1 and rmOC90 modulated crystals as calcite (Figure 5), which is consistent with the polymorph of CaCO_3_ observed in normal otoconia. The propagation of steps on single crystal surfaces of calcite and other biomineral phases and the impact of peptides and proteins on that process have been explored in great detail over fifteen years [25], [26], [27], [28], [29], [30], [31], [32], [33]. The salient point of those studies with respect to the current work is that soluble peptides and proteins often produce differential inhibition of calcite steps due to step-specific binding affinity that result in shape modification. The acute steps are often more susceptible to inhibition and induce an elongation of the crystals along the crystallographic (001) direction with rounding (hk0) faces. However, many acidic peptides and proteins also exhibit differential acceleration of calcite step growth at low concentrations. Although the mechanism of acceleration has never been proven, analyses of the dependence on concentration and hydrophobicity suggest these peptides and proteins act as catalysts to increase calcium desolvation rates.

Given the findings of these previous studies, the results presented here indicate that rmOC90 is a soluble protein that adsorbs to calcite steps and blocks calcium or carbonate ions from attaching to the kink sites [32], [34] and reduces the step speed at low concentrations as seen in Figure 6a. Moreover, it binds preferentially to the acute steps, producing the morphology seen in Figure 3B with stabilization of the side faces perpendicular to the c-axis, consistent with the results of Lu et al. [11] and results observed for other acidic proteins [29]. In contrast, rhOtolin-1 in the dissolved state enhances solute attachment rates and does so preferentially at the obtuse steps, leading to an elongation along the c-axis of calcite. Most importantly, when the preferential acceleration of the obtuse steps by rhOtolin-1 is combined with the preferential inhibition of the acute steps by rmOC90, the natural outcome would be a spindle shaped crystal with well-defined obtuse-obtuse edges at the (001) extremities demonstrated in Figure 3D. Moreover because rhOtolin-1 forms a fibrillar network on the mica surfaces, it is likely to be a matrix former.

Taken together these findings suggest that Otolin-1 acts as a matrix protein, as its collagen family implies, and as a modulator of calcite crystal morphology via its direct effect on crystal growth kinetics of the calcite crystals and via the synergistic potentiation of OC90.
